# Activating Mutations in β-Catenin in Colon Cancer Cells Alter Their Interaction with Macrophages; the Role of Snail

**DOI:** 10.1371/journal.pone.0045462

**Published:** 2012-09-21

**Authors:** Pawan Kaler, Leonard Augenlicht, Lidija Klampfer

**Affiliations:** Department of Oncology, Albert Einstein Cancer Center, Montefiore Medical Center, Bronx, New York, United States of America; National Cancer Center, Japan

## Abstract

**Background:**

Tumor cells become addicted to both activated oncogenes and to proliferative and pro-survival signals provided by the abnormal tumor microenvironment. Although numerous soluble factors have been identified that shape the crosstalk between tumor cells and stroma, it has not been established how oncogenic mutations in the tumor cells alter their interaction with normal cells in the tumor microenvironment.

**Principal Findings:**

We showed that the isogenic HCT116 and Hke-3 cells, which differ only by the presence of the mutant kRas allele, both stimulate macrophages to produce IL1β. In turn, macrophages enhanced Wnt signaling, proliferation and survival in both HCT116 and Hke-3 cells, demonstrating that signaling by oncogenic kRas in tumor cells does not impact their interaction with macrophages. HCT116 cells are heterozygous for β-catenin (HCT116^WT/MT^), harboring one wild type (WT) and one mutant (MT) allele, but isogenic lines that carry only the WT (HCT116^WT^) or MT β-catenin allele (HCT116^MT^) have been generated. We showed that macrophages promoted Wnt signaling in cells that carry the MT β-catenin allele, but not in HCT116^WT^ cells. Consistent with this observation, macrophages and IL1β failed to stabilize Snail in HCT116^WT^ cells, and to protect these cells from TRAIL-induced apoptosis. Finally, we demonstrated that HCT116 cells expressing dominant negative TCF4 (dnTCF4) or HCT116 cells with silenced Snail failed to stimulate IL1β production in macrophages, demonstrating that tumor cells activate macrophages via a Wnt-dependent factor.

**Significance:**

Our data demonstrate that oncogenic β-catenin mutations in tumor cells, and subsequent activation of Wnt signaling, not only trigger cell-intrinsic alterations, but also have a significant impact on the crosstalk of tumor cells with the tumor associated macrophages.

## Introduction

Beta catenin (β-catenin) is an E-cadherin binding protein and thus plays an important role in cell-cell adhesion [1]. In addition, it acts as an effector of Wnt signaling [2]. In the absence of Wnt signaling β-catenin is phosphorylated by GSK3β, resulting in its ubiquitination and subsequent degradation in the proteasome [3]. The activity of β-catenin is controlled by the tumor suppressor Adenomatous Polyposis Coli (Apc) and inactivating mutations in the Apc gene, which prevent β-catenin degradation, are found in the large majority of sporadic colorectal cancers [4], [5]. Further, approximately 10% of colorectal cancers carry mutations in the GSK3β phosphorylation site located in the N-terminus of β-catenin [6]. Apc mutations and β-catenin mutations are mutually exclusive in colon cancer, as they both lead to stabilization of β-catenin and in constitutive β-catenin/TCF transcriptional activity.

HCT116 cells are heterozygous for β-catenin, harboring one wild type (WT) allele and one mutant (MT) allele with inactivation of SER45, one of the residues phosphorylated by GSK3β that is frequently mutated in tumors [6], [7]. Isogenic HCT116 cell lines that carry only WT or MT allele have been generated to study the role of oncogenic β-catenin signaling in colon cancer [8], [9]. As expected, deletion of the MT β-catenin allele in HCT116 cells significantly reduced β-catenin/TCF mediated transcriptional activity in these cells. While the total levels of β-catenin were similar in cells with disrupted WT or MT β-catenin allele, inactivation of the MT allele resulted in redistribution of β-catenin to cell membranes associated with cell junctions [8], [9]. Surprisingly, expression of typical Wnt target genes, such as c-myc, was not reduced upon deletion of the mutant β-catenin allele, and growth characteristics of these cells under standard conditions were not significantly affected *in vitro* [8]. These results suggested that mutations in β-catenin, in addition to initiating a plethora of cell intrinsic alterations, may also affect the interaction of tumor cells with the components of the tumor microenvironment and thus contribute to tumor development *in vivo*.

We recently reported that tumor cell-derived factor(s) activated macrophages to produce IL1β, which in turn inactivated GSK3β, increased the levels of unphosphorylated β-catenin and stimulated Wnt signaling in HCT116 cells [10]. We showed that macrophage-derived factors stimulated Wnt signaling in an NF-κB-dependent manner [11]. Consistent with this, constitutive activation of IKK2 in intestinal epithelial cells, which is sufficient to induce intestinal tumors in mice, triggers mobilization of macrophages and the expression of a variety of proinflammatory cytokines, including TNF and IL1β [12], and cells with constitutive activation of IKK2 displayed activated Wnt signaling.

**Figure 1:**
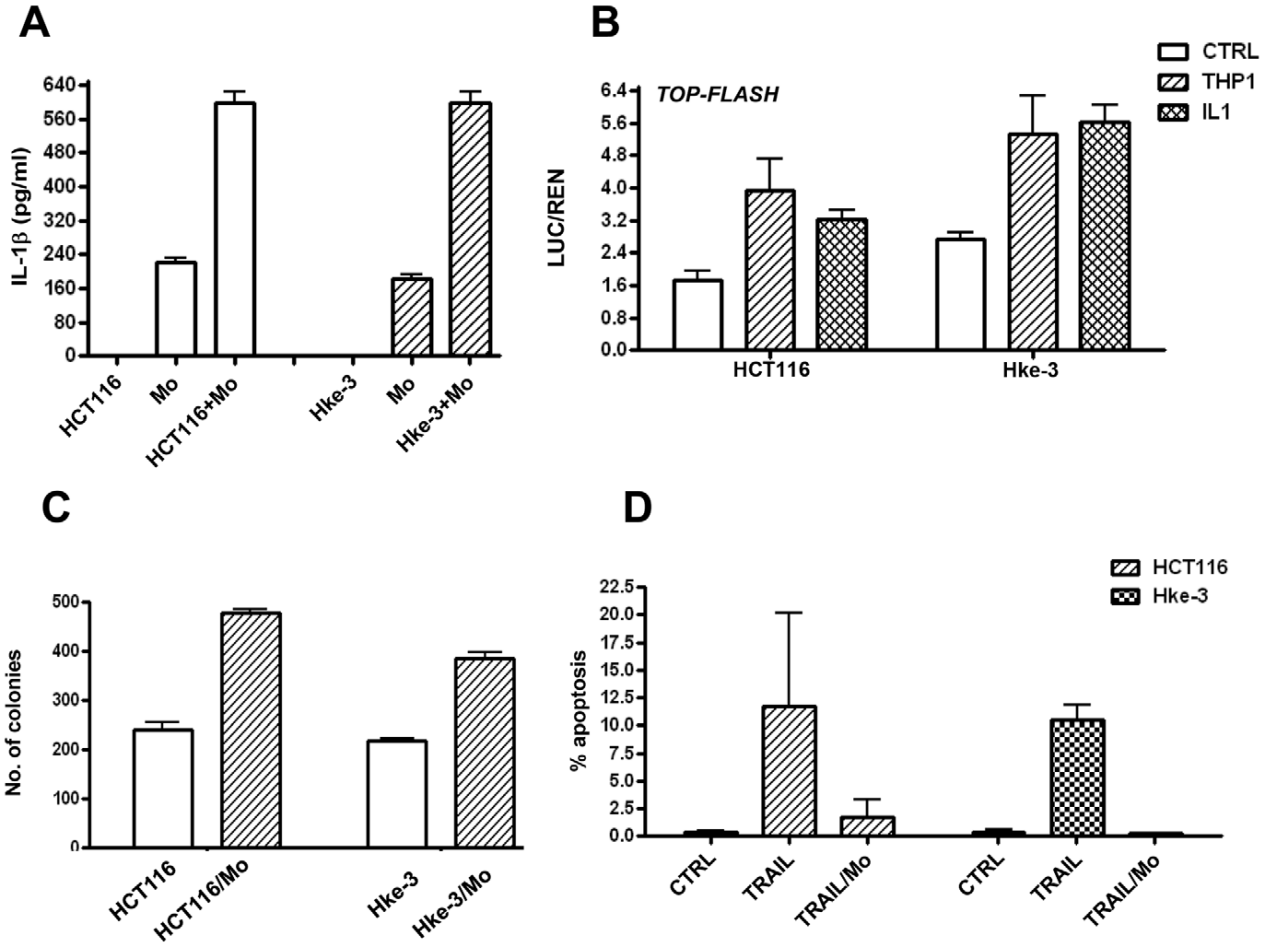
The presence of the mutant kRas in tumor cells does not alter their interaction with macrophages. A: HCT116 and Hke-3 cells were compared for their ability to induce IL1β in peripheral blood monocytes (Mo). The ability of THP1 macrophages and IL1β to induce Wnt signaling in HCT116 and HKe-3 cells (B), to promote clonogenic growth (C) and to protect from TRAIL-induced apoptosis (D) was measured.

We showed that macrophage-derived factors stabilize Snail in colon cancer cells [13], a transcription factor that promotes the invasive and metastatic phenotype through induction of an epithelial mesenchymal transition (EMT) [14]. Snail is a Wnt target gene [15], [16], but it has also been shown to interact with β-catenin and to co-stimulate Wnt target genes [15]–[17], indicating an intricate interplay between Wnt signaling and Snail. In addition, Snail regulates growth and survival of tumor cells, and has been linked to the formation of cancer stem cells [18]. Increased Wnt signaling and stabilization of Snail therefore strongly suggest that macrophage-derived factors increase the stem cell properties of cancer cells, which contribute to metastasis and display resistance to therapy. Consistent with this, we showed that macrophage-derived factors protected tumor cells from TRAIL-induced apoptosis [13].

**Figure 2:**
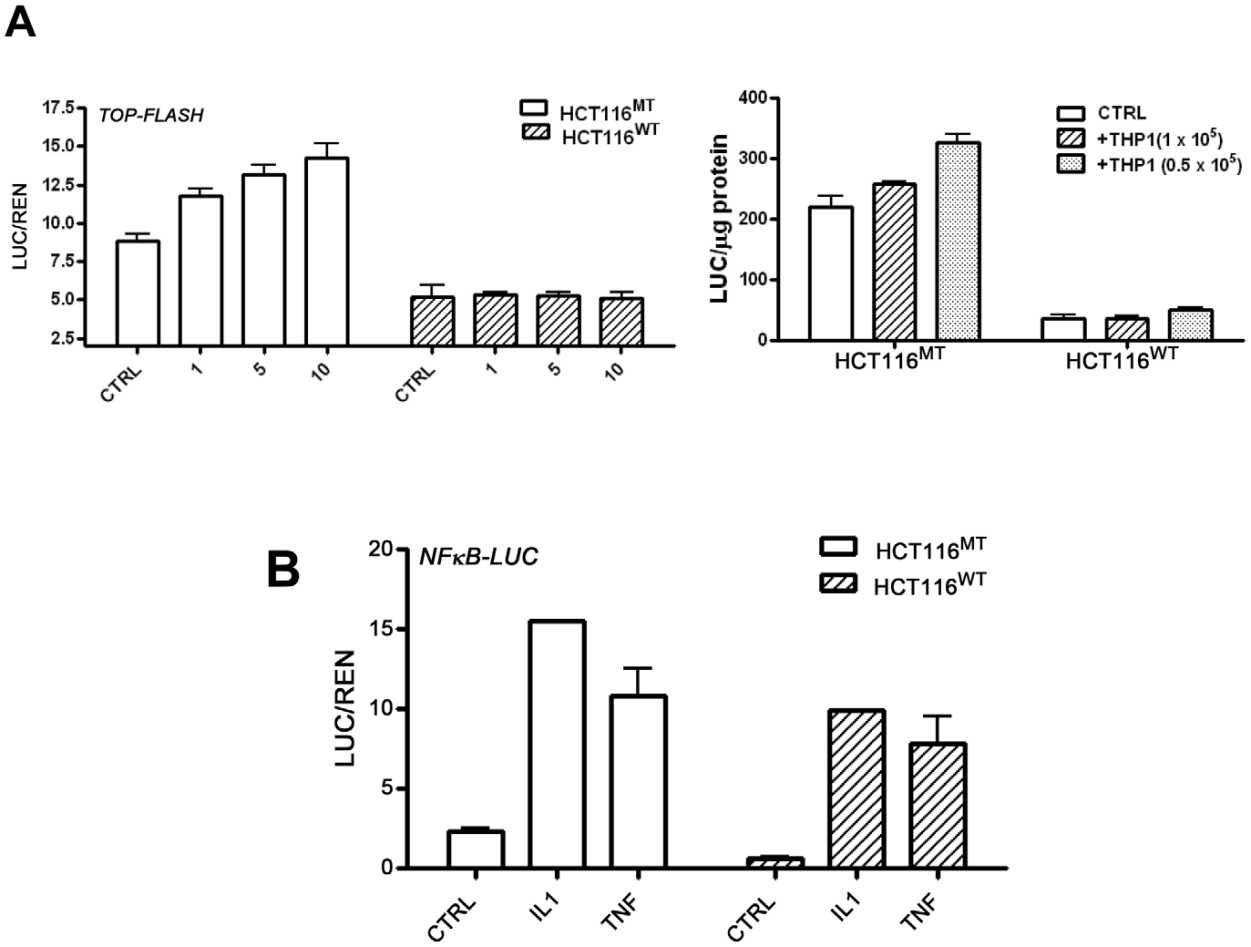
Interaction of HCT116 cells with macrophages is altered by mutations in β-catenin. A: Isogenic HCT116 cell lines with the deleted WT (HCT116^MT^) or MT β-catenin allele (HCT116^WT^) were transfected with the TOP-LUC reporter gene and were treated with increasing concentrations of IL1β or were cultured with THP1 macrophages as indicated. B: HCT116 cells were transfected with the NF-κB-reporter gene and were treated with TNF and IL1β as indicated.

Here we show that the ability of HCT116 cells to stimulate macrophages is completely dependent on the presence of the endogenous mutant β-catenin and subsequent stabilization of Snail in epithelial cells, corroborating our hypothesis that acquisition of oncogenic mutations in intestinal epithelial cells alters their interaction with their microenvironment. We showed that colon cancer cells stimulate macrophages through the product of a Wnt target gene. Our data suggest that Snail regulates the expression of this gene and thus plays a crucial role in the crosstalk between colon cancer cells and macrophages.

**Figure 3:**
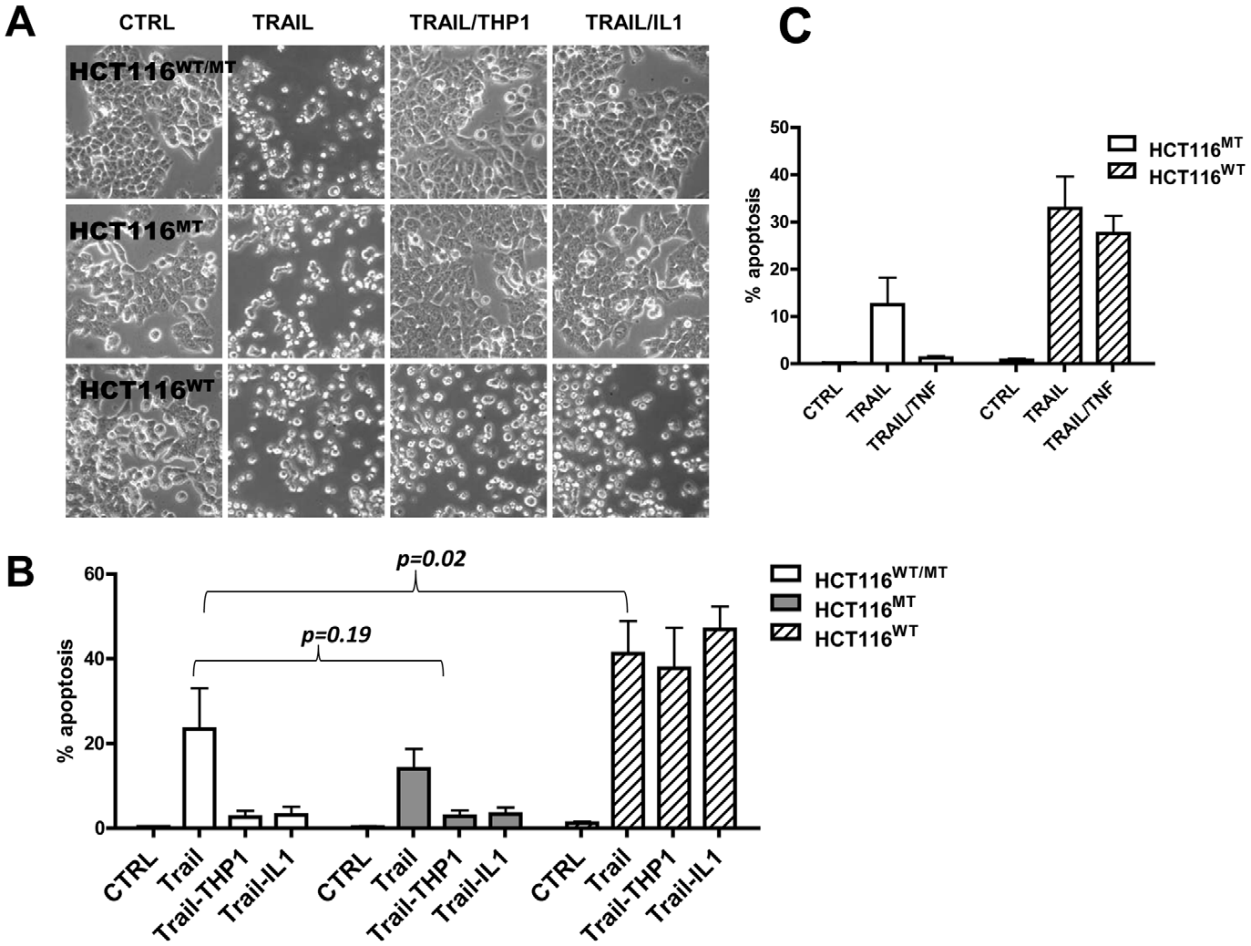
Macrophage-derived factors do not protect HCT116^WT^ cells from TRAIL induced apoptosis. A: Parental HCT116 cells or HCT116 with deleted WT or MT β-catenin allele were treated with TRAIL in the absence of THP1 macrophages or IL1 as indicated. B: The extent of apoptosis was determined in 4 independent experiments. C: HCT116^WT^ and HCT116^MT^ cells were treated with TRAIL in the absence or the presence of TNF and the extent of apoptosis was determined by PI staining.

## Results

### Mutations in β-catenin, but not kras, alter colon cancer cell interactions with macrophages

We reported that colon cancer cells, via a soluble factor, activate macrophages to secrete IL1β, which in turn promotes β-catenin/TCF4 transcriptional activity in the cancer cells and stimulates their growth [10]. To establish whether acquisition of the oncogenic kRas mutation in tumor cells alters their interaction with macrophages, we performed experiments in HCT116 and Hke-3 cells, isogenic cell lines that differ only by the presence of the mutant kRas allele [19]. We showed that both HCT116 and Hke-3 cells induced IL1β in peripheral blood monocytes, precursors of the tumor associated macrophages (Fig. 1A). Accordingly, macrophages and IL1β stimulated Wnt signaling (Fig. 1B), promoted growth (Fig. 1C) and protected HCT116 and Hke-3 cells from TRAIL-induced apoptosis (Fig. 1D). These studies demonstrated that the presence of oncogenic kRas in tumor cells does not have a significant impact on their interaction with tumor associated macrophages.

**Figure 4:**
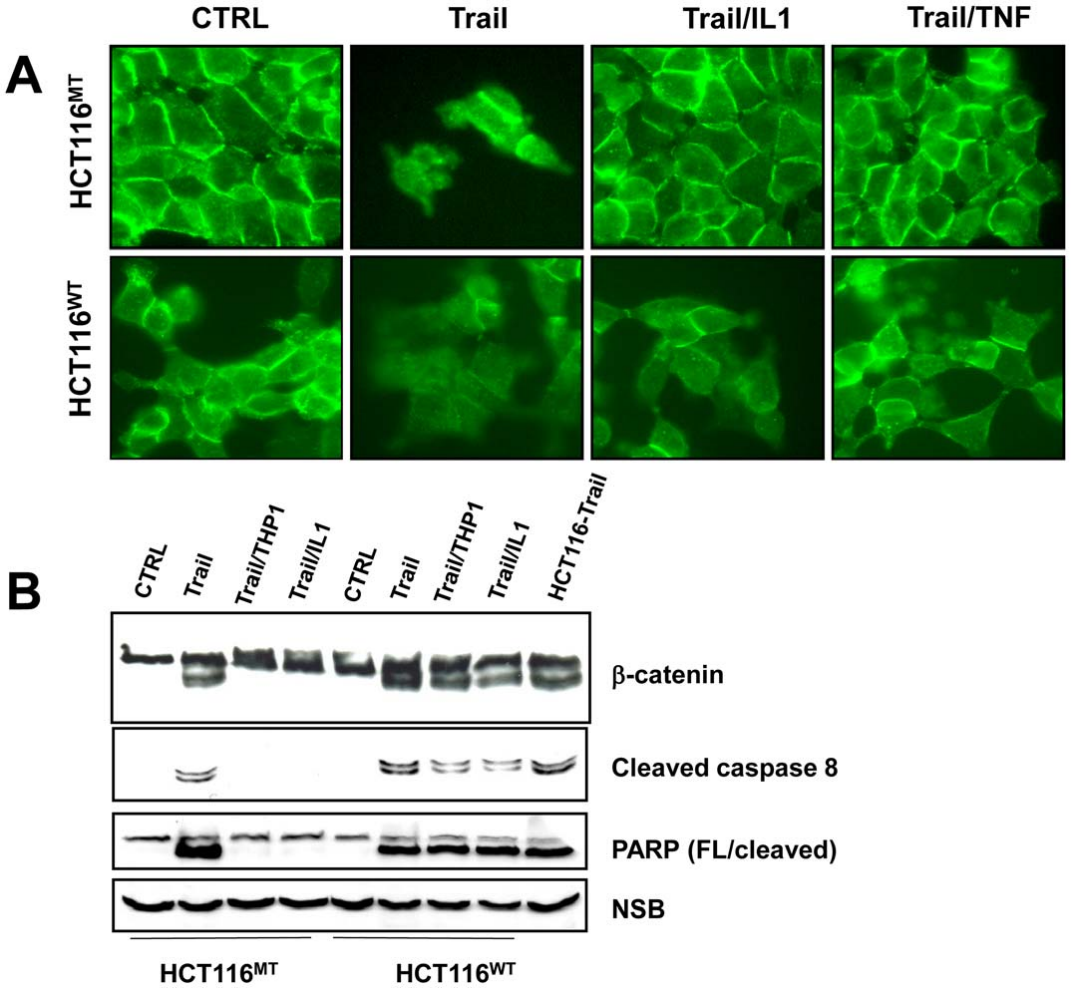
Macrophage-derived factors do not protect from TRAIL induced apoptosis in the absence of the mutant β-catenin allele. A: B: HCT116^WT^ and HCT116^MT^ cells were treated with TRAIL in the absence or the presence of IL1β as indicated and the localization of β-catenin was determined by immunofluorescence in cells treated with TRAIL in the absence or the presence of IL-1β or TNF as indicated. B: The extent of caspase 8 activation and the cleavage of PARP and β-catenin were determined by immunoblotting.

HCT116 cells are also heterozygous for β-catenin, containing one wild type (WT) allele and one mutant (MT) allele with a 3-bp deletion that eliminates the serine residue at codon 45 [7], resulting in β-catenin stabilization and activation of Wnt signaling. To determine whether the presence of the activated, MT β-catenin in tumor cells modulates their interaction with macrophages we performed experiments in HCT116 cells with a deletion of either the WT (HCT116^MT^) or MT β-catenin allele (HCT116^WT^) [8]. The isogenic cell lines were treated with IL1β or were cultured with THP1 macrophages. As in parental HCT116 cells, macrophages and IL1β induced Wnt signaling in HCT116^MT^ cells (Fig. 2A). In contrast, HCT116^WT^ cells, which showed lower Wnt signaling, failed to respond to IL1β or to macrophages with increased Wnt signaling (Fig. 2A). The ability of IL1β and TNF to activate NF-κB, the major signaling pathway activated by these cytokines, was not affected by the status of β-catenin (Fig. 2B), demonstrating pathway specificity, and is consistent with our previous finding that NFκB signaling is upstream of Wnt signaling [11]. These results demonstrated that the presence of the MT β-catenin allele in tumor cells, which results in constitutive β-catenin/TCF4 activity, alters their interaction with macrophages.

**Figure 5:**
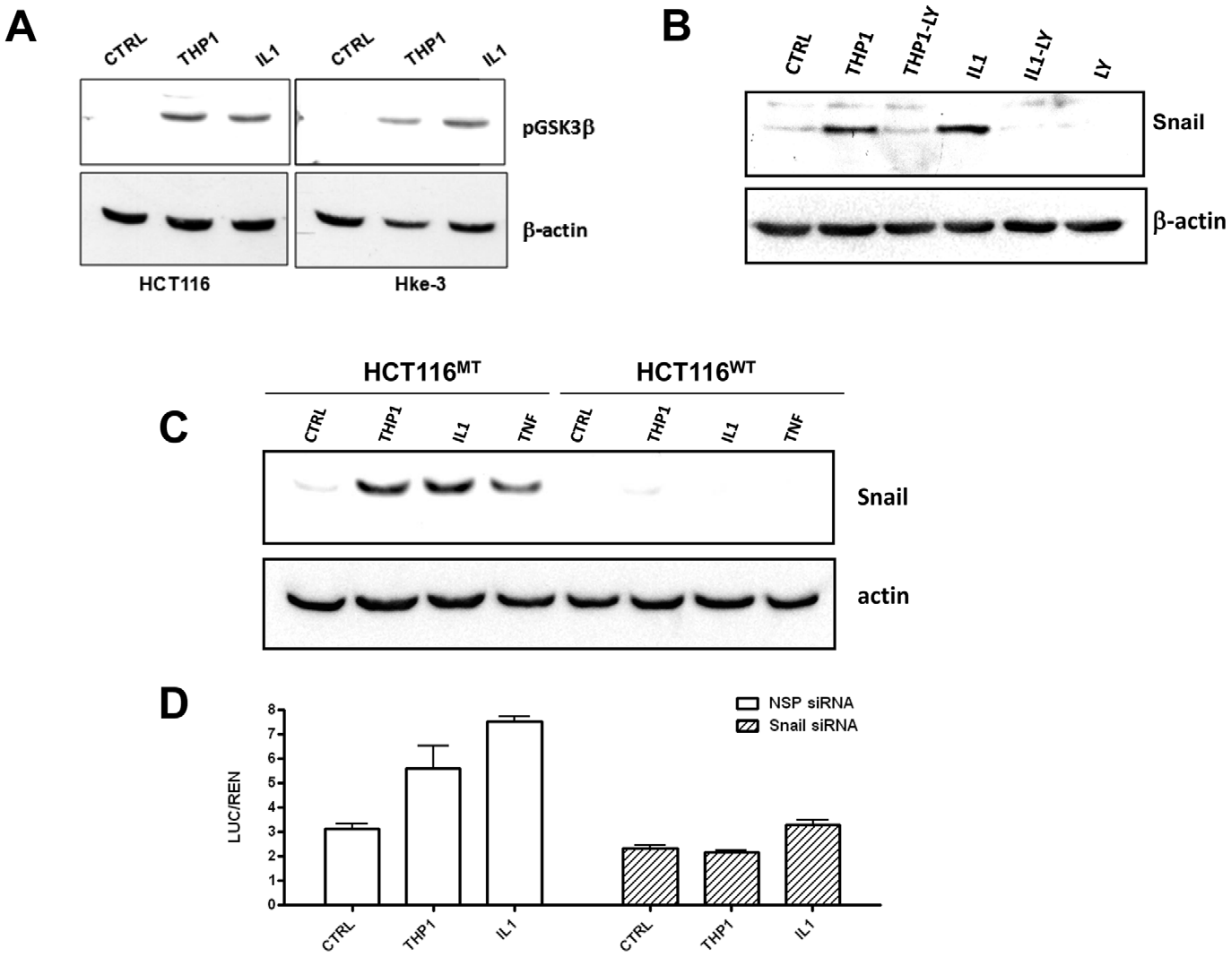
Snail is stabilized in cells that harbor the mutant β-catenin allele. A: Macrophages and IL1β inactivate GSK3β activity in HCT116 and Hke-3 cells. B: The levels of Snail were determined in HCT116 cells that were cultured with THP1 macrophages or were stimulated with IL1β in the absence or the presence of LY294002 as indicated. C: HCT116^WT^ and HCT116^MT^ cells were cultured with THP1 macrophages or were treated with IL1β or TNF as indicated for 24 hours. D: The ability of THP1 macrophages and recombinant IL1β to induce Wnt signaling was compared in cells transfected with nonspecific (NSP) or Snail specific siRNA.

### Macrophages fail to protect HCT116 cells that lack the MT β-catenin allele from TRAIL induced apoptosis

We demonstrated that macrophage-derived factors protect colon cancer cells from TRAIL-induced apoptosis through a mechanism dependent on Wnt signaling [13], suggesting that HCT116^WT^ cells (lacking the mutant β-catenin allele), which did not respond to macrophages with enhanced Wnt signaling (Fig. 1), may display an altered response to TRAIL. We treated the parental HCT116 cells, HCT116^WT^ and HCT116^MT^ cells with TRAIL in the absence or the presence of THP1 macrophages or IL1β. As shown in Fig. 3, HCT116 cells that lack the mutant β-catenin allele (HCT116^WT^) displayed increased sensitivity to TRAIL-induced apoptosis, which is consistent with the fact that Wnt signaling protects cells from TRAIL-induced apoptosis [20]. In contrast to parental HCT116 cells and HCT116 cells with deleted WT β-catenin allele (HCT116^MT^), HCT116^WT^ cells were not protected from TRAIL-induced apoptosis by macrophage, IL1β (Fig. 3A, B) or by TNF (Fig. 3C).

**Figure 6:**
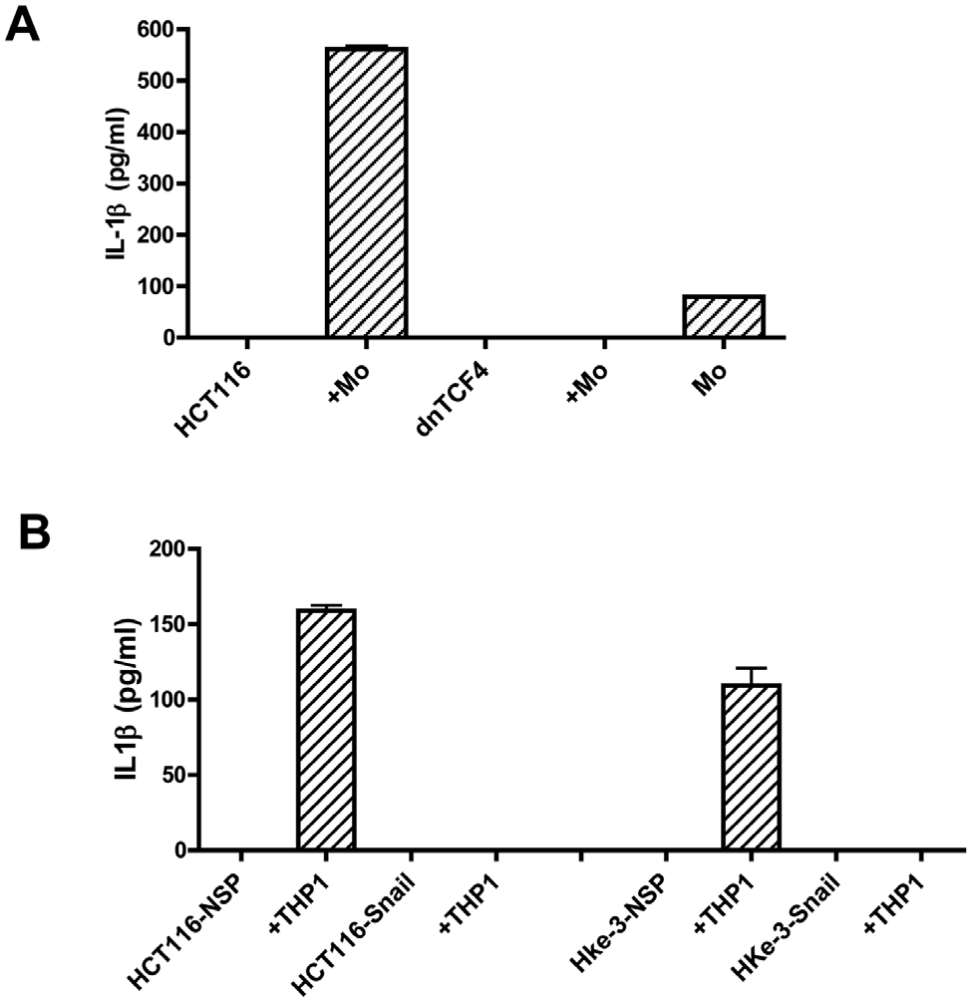
Wnt signaling in HCT116 cells is required for stimulation of monocytes. A: HCT116 transfected with an empty vector or dnTCF4 were co-cultured with primary peripheral blood monocytes (Mo) for 24 hours and the amount of IL1β was determined by ELISA. B: HCT116 or Hke-3 cells transfected with NSP (nonspecific) or Snail specific RNAi were cultured with THP1 monocytes and the amount of IL1β was determined by ELISA.

Despite harboring mutant β-catenin HCT116 cells display predominantly plasma membrane localization of β-catenin [8]. Treatment of cells with TRAIL triggered the loss of distinct membrane staining in both HCT116^MT^ and HCT116^WT^ cells (Fig. 4A), consistent with the cleavage of β-catenin in TRAIL-treated cells (Fig. 4B). However, while TNF and IL1β restored membrane localization of β-catenin in HCT116^MT^ cells, they failed to counteract the effect of TRAIL in HCT116^WT^ cells (Fig. 4A). Accordingly, macrophages and IL1β failed to inhibit TRAIL-induced cleavage of β-catenin, activation of caspase-8, and subsequent processing of PARP in HCT116^WT^ cells (Fig. 4B).

**Figure 7:**
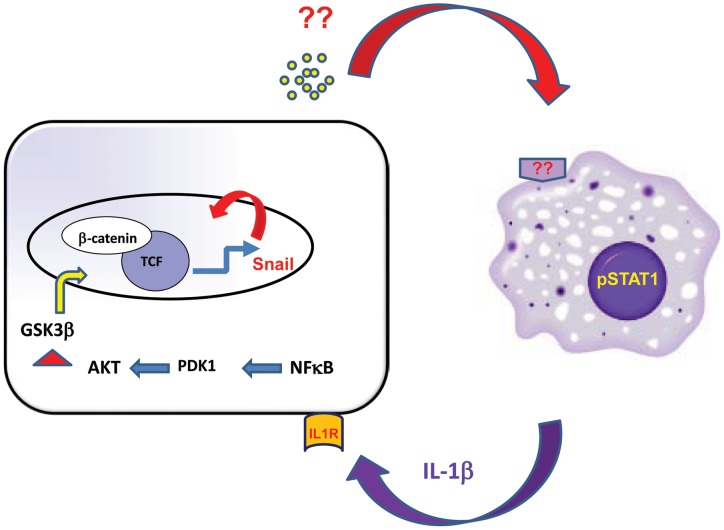
A proposed model for the interaction of tumor cells with macrophages: Colon cancer cells produce a soluble factor, a product of a Wnt regulated gene, which through binding to an unknown receptor stimulates macrophages to release IL1β. IL1β in turn binds IL1R on tumor cells and stimulates Wnt signaling. We showed that macrophage-derived factor stabilize Snail in tumor cells, which further promotes Wnt signaling and is required for the ability of tumor cells to activate macrophages to produce IL1β.

These data confirmed that cells with active Wnt signaling display an altered response to macrophage-derived factors, potentially affecting their susceptibility to therapeutic agents.

### Lack of Snail stabilization in HCT116 cells with inactivated MT β-catenin allele

Wnt signaling has been shown to promote the transcription, the stability and nuclear localization of Snail [15], [16]. Indeed, we showed that inactivation of GSK3β by LiCl or by a specific GSK3β inhibitor, AR-A014418, was sufficient to increase the levels of Snail in HCT116 cells [13]. We showed that overexpression of Snail promotes TCF4-β-catenin-driven transcription, and that silencing of Snail interferes with Wnt signaling in HCT116 cells [13], consistent with the ability of Snail to associate with β-catenin and to co-regulate the expression of Wnt target genes [17], [21].

Macrophages and IL1β inactivate GSK3β in both HCT116 and Hke-3 cells (Fig. 5A) and, consistently, stabilize Snail independently of the presence kRas mutations in tumor cells [13]. Activation of GSK3β by a specific PI3K inhibitor, YL294002, precluded macrophage- and IL1β- induced stabilization of Snail in HCT116 cells, confirming that macrophage-derived factors stabilize Snail through their ability to inactivate GSK3β (Fig. 5B).

We showed earlier that macrophage-derived factors failed to stabilize Snail in HCT116 cells expressing dnTCF4, consistent with the notion that active Wnt signaling is required for stabilization of Snail [13]. Here we compared the ability of macrophage-derived factors to stabilize Snail in HCT116 cells that had a deletion of either the WT or the MT β-catenin allele. As in parental HCT116 cells, macrophages, IL1β and TNF stabilized Snail in HCT116^MT^ cells (Fig. 5C). In contrast, macrophage-derived factors failed to stabilize Snail in HCT116^WT^ cells (Fig. 5C), consistent with the inability of macrophages to enhance Wnt signaling in these cells (Fig. 1). We silenced Snail in HCT116 cells and confirmed that, consistent with our published data [13], macrophages and IL1β failed to stimulate Wnt signaling in the absence of Snail (Fig. 5D).

### Tumor cells activate macrophages via a Wnt-dependent factor

We demonstrated that tumor-derived factor(s) stimulate macrophages to secrete a variety of soluble factors, including IL1β, a cytokine that we established was required for the crosstalk between tumor cells and macrophages [10]. We showed that HCT116 cells expressing dnTCF4 failed to stimulate peripheral blood monocytes to secrete IL1β (Fig. 6A), confirming that Wnt signaling is required for the expression of the tumor derived factor that stimulates macrophages.

Likewise, silencing of Snail, a Wnt target gene, in HCT116 or Hke-3 cells resulted in inability of these cells to trigger IL1β production in transformed THP1 monocytes (Fig. 6B). Together, these data confirmed that colon cancer cells activate macrophages via a Wnt/Snail-regulated factor, which is required for the production of IL1β.

## Discussion

Although appropriately stimulated macrophages have the inherent ability to kill tumor cells, it has become clear that macrophages recruited to the tumor microenvironment and shaped by tumor-derived factors lose the ability to destroy tumor cells. Moreover, it has been established that tumor associated macrophages (TAMs) provide soluble factors that promote the growth, survival and metastatic spread of colon cancer cells [22]–[24]. Several macrophage-derived factors have been identified that regulate the growth of tumor cells. We showed that macrophage-derived IL1β promotes Wnt signaling in colon cancer cells and thus regulates their growth and survival [10], [13]. In turn, cancer cells secrete factors that attract and activate myeloid cells, and thus propagate a self-amplifying loop that sustains tumor growth. However, much less is known about the nature of the tumor-derived factors that blunt the cytotoxic ability of macrophages and stimulate their tumor promoting properties. Tumor-derived CCL2 promotes the recruitment of inflammatory monocytes and [25], and tumor-derived versican has been shown to activate myeloid cells via TLR2-dependent signaling [26].

We showed that macrophage-induced activation of Wnt signaling in colon cancer cells promotes their growth and survival, consistent with a prominent role of Wnt signaling in colon cancer, and recurrent mutations of Apc or β-catenin in the majority of sporadic colon cancers [27]. In this study we demonstrated that activation of Wnt signaling in colon cancer cells not only initiates a plethora of cell-intrinsic alterations in epithelial cells, but also impacts how epithelial cells communicate with macrophages. Inactivation of the MT β-catenin allele in HCT116 cells significantly altered their interaction with macrophages. We showed that macrophages failed to promote Wnt signaling and to protect HCT116^WT^ cells from TRAIL-induced apoptosis. This is consistent with our earlier report that expression of dnTCF4 in tumor cells prevented signaling between macrophages and tumor cells [11], [13] and confirms that acquisition of oncogenic mutations alters the interaction of epithelial cells with the adjacent stroma.

Like IL1β, FGF19 has been shown to modulate Wnt signaling in HCT116 cells [28]. However, in contrast to our finding, FGF19 increased β-catenin/TCF transcriptional activity only in parental HCT116 cells and in HCT116 cells that retained the WT β-catenin allele [28]. Together these data underscore the importance of β-catenin mutations for the response of tumor cells to autocrine and paracrine signals and thus for their interaction with cells in the tumor microenvironment. Our data confirmed that activation of oncogenic pathways, such as Wnt, not only triggers cell-intrinsic changes, but also induces stromal alterations that are required for tumor progression. In contrast to β-catenin mutations, oncogenic activation of kRas in intestinal epithelial cells did not impact the crosstalk between epithelial cells and stroma.

We showed that macrophage-derived factors stabilize Snail in colon cancer cells [13], which regulates epithelial mesenchymal transition and thereby promotes stemness of cancer cells and their ability for metastatic seeding [29],[30]. Snail is a Wnt target gene [15], [16] which, in turn, interacts with β-catenin, and thus co-regulates the expression of Wnt target genes [17]. In contrast to the parental HCT116 cells, macrophage-derived factors failed to stabilize Snail in HCT116 cells with deleted MT β-catenin allele (Fig. 5C). We demonstrated that the ability of macrophages and IL1β to inhibit TRAIL-induced apoptosis and to promote clonogenic growth of tumor cells was also inhibited in tumor cells with silenced Snail expression [13], demonstrating that Snail regulates several steps in colon cancer progression and pointing to a crucial role of Snail in the crosstalk between tumor cells and macrophages. Here we show that active Wnt signaling in colon cancer cells, and Wnt-dependent stabilization of Snail specifically, are required for tumor cells to stimulate macrophages to produce IL1β (Fig. 6). Similarly, recent findings demonstrated that Snail upregulates the expression of proinflammatory mediators in oral keratinocytes, including IL6, IL8, CXCL1 and IL1β [31]. This is consistent with our finding that macrophage-derived factors protect colon cancer cells from TRAIL-induced apoptosis through stabilization of Snail [13]. Therefore, the lack of Snail stabilization in HCT116 cells with inactivated MT β-catenin allele underlies the inability of macrophages to protect these cells from TRAIL-induced apoptosis. These data confirmed that activation of Wnt signaling and subsequent stabilization of Snail in colon cancer cells significantly impacts their interaction with macrophages, and suggests a pivotal role of Snail in the crosstalk between colon cancer cells and macrophages.

Together, our data strongly suggest that tumor cells stimulate macrophages via a Wnt-dependent factor. This is consistent with our finding that HCT116 cells expressing dnTCF4 failed to stimulate IL1β in macrophages (Fig. 6) and thus exhibit perturbed crosstalk with macrophages [13]. Indeed, we showed that HCT116 cells expressing dnTCF4 have increased response to TRAIL, and that macrophages do not protect these cells from TRAIL-induced apoptosis [13]. Experiments are underway to identify the tumor-derived factors which stimulate macrophages, and to determine whether the expression of Toll-Like Receptors (TLRs) on macrophages is required for the crosstalk between colon cancer cells and macrophages (Figure 7).

Human colon tumors display heterogeneous levels of Wnt activity [32] and it has been shown that only cells with high levels of Wnt signaling display colon cancer stem cell (CSC) properties [33], [34]. Indeed, silencing of β-catenin in HCT116 cells has been shown to decrease the number of colonospheres, which are highly enriched in CSCs [35]. Consistent with this, HCT116^WT^ cells failed to form spheroids when plated in low serum conditions [9]. We demonstrated that macrophages and IL1β enhanced Wnt signaling and stabilized Snail in tumor cells [13], which has been shown to endow cells with stem cell-like properties [18], suggesting that macrophage- derived factors contribute to heterogeneity of colon tumors by expanding the fraction of tumor cells that have cancer stem cell properties. Likewise, myofibroblast-derived factors have been shown to impose the CSC phenotype by promoting Wnt signaling in tumor cells [34] and periostin, a component of the extracellular matrix, promotes the stemness of cancer cells and increases their ability to form metastasis by upregulating Wnt signaling [36].

Because an active Wnt signaling is a signature of colon cancer stem cells, it is possible that cancer stem cells have an exclusive ability to interact with the cells in the tumor microenvironment. Consistent with this, cancer associated fibroblasts promote the invasiveness of CD133^+^ colon cancer stem cells more efficiently than that of CD133^−^ cells [37]. This is likely to contribute to the unique ability of cancer stem cells to propagate tumor growth and to confer resistance to therapeutic approaches.

## Materials and Methods

### Cell lines and co-culture experiments

The HCT116 and Hke-3 colorectal carcinoma cell lines, which differ only by the presence of the mutant k-Ras allele [19], were cultured in MEM, supplemented with 10% FCS. HCT116 with disrupted WT or MT β-catenin allele were provided by Kenneth Kinzler [8]. The human monocytic cell line, THP1, was cultured in RPMI. Normal human monocytes, >90% CD14 and CD11c positive and less than 1% anti T cell receptor positive, were purchased from *Astarte Biologics* (Redmond, WA). Tumor cells and monocytes/macrophages were co-cultured separated by transwell inserts of a polycarbonate membrane with 0.4 μM pore size, which preclude direct cell-cell contact, but permit the exchange of soluble factors (Corning Incorporated, Lowell, MA).

For clonogenic assays, HCT116 and Hke-3 cells were seeded at a density of 200 cells per well of a six well plate alone or together with peripheral blood monocytes for 7 days as described previously [10], [11]. Colonies were fixed and stained with 6% glutaraldehyde and 0.5% crystal violet and counted using Total Lab 1.1 software (Nonlinear Dynamics, Durham, NC, USA).

### Apoptosis assay

Cells were treated with recombinant TRAIL (50 ng/ml, the optimal concentration for the induction of apoptosis in HCT116 cells) alone or in the presence of macrophages, IL1β (5 ng/ml) or TNFα (10 ng/ml) for 7 hours. Cells were resuspended in hypotonic buffer (0.1% Triton X-100, 0.1% sodium citrate) and stained with propidium iodide (50 μg/ml) for 4 hours at 4°C as described [38]. Samples were analyzed by flow cytometry and cell cycle distribution and the extent of apoptosis (cells with a subG1 DNA content) were analyzed by the *Modfit* software. Cells were treated with TRAIL for ∼7 hours and were collected for evaluation based on morphological criteria. We confirmed the apoptotic nature of cells by biochemical analysis of cell lysates. Statistical analysis was performed using unpaired Student's t test, with values <0.05 considered statistically significant.

### Transient transfection and Reporter gene assay

HCT116 and Hke-3 cells were transiently transfected with the TOP-FLASH plasmid (carrying a TCF specific promoter) or the TOP-FOP plasmid (a control plasmid carrying a mutated promoter) using the calcium phosphate method. Transfection efficiency was normalized by co-transfection with pTK-Renilla and luciferase activity was determined according to the vendor's protocol (Dual Luciferase reporter assay, Promega, Madison, WI).

### Immunofluorescence

For the subcellular detection of β-catenin by immunofluorescence, cells were fixed in 4% paraformaldehyde for 30 minutes. The cells were incubated with anti-β-catenin antibody (BD Biosciences, San Jose, CA, 1∶100) for 1 h at 37°C and with secondary anti-rabbit antibody conjugated to FITC for 45 min at 37°C. Images were acquired with a SPOT CCD camera and analyzed by SPOT software.

### Western Blot

Western blots were performed using standard procedures. Membranes were blocked with 5% milk in TBS containing 0.1% Tween 20, and incubated with antibodies specific for caspase 8 (Abcam), caspase 9, PARP and Snail (Cell Signaling Technology), pGSK3 (Millipore, Billerica, MA), total β-catenin (BD Biosciences, San Jose, CA), and β-actin (Sigma Aldrich, St. Louis, MO). Immunoreactive bands were visualized by chemiluminescence (Amersham ECL^TM^ western blotting detection kit, Piscataway, NJ).
